# Montreal Cognitive Assessment Hearing Impairment (MoCA-H) in Brazilian Portuguese: criterion and construct validity

**DOI:** 10.1590/2317-1782/e20240249en

**Published:** 2025-10-13

**Authors:** Gabriela Konrath, Rochele Martins Machado, Karina Carlesso Pagliarin, Fernanda Soares Aurélio Patatt

**Affiliations:** 1 Departamento de Fonoaudiologia, Universidade Federal de Santa Maria – UFSM - Santa Maria (RS), Brasil.

**Keywords:** Hearing, Hearing Loss, Cognitive Dysfunction, Aging, Presbycusis, Psychometrics, Validation Study, Mental Status and Dementia Tests

## Abstract

**Purpose:**

To find evidence of criterion and construct validity for the Montreal Cognitive Assessment Hearing Impairment (MoCA-H) protocol in Brazilian Portuguese.

**Methods:**

The sample consisted of 70 elderly people divided into two groups: Group 1-50 subjects with hearing loss and no cognitive decline; Group 2-20 subjects with hearing loss and cognitive decline. Criterion validity was obtained by comparing Group 1 and 2 considering the overall score and the eight domains assessed in the MoCA-H. The data were analyzed using the Mann-Whitney U-test and Student's T-test, respecting the characteristics of the data collected. To verify construct validity, the correlation between the total scores of the Mini-Mental State Examination (MMSE) and the MoCA-H obtained by Group 2 was analyzed. Spearman's Correlation Test was used for this purpose.

**Results:**

The analysis of criterion validity showed a difference between the groups with and without decline in naming, attention, language, abstraction, memory and delayed recall skills, as well as the MoCA-H total score, indicating significantly higher performance of Group 1. The construct validity correlation analysis was weak and non-significant (Rho=0.384; p=0.095) between the MoCA-H and MMSE scores.

**Conclusion:**

The MoCA-H protocol showed good criterion validity for this specific population, making it a reliable tool for screening mild cognitive decline. However, it did not show satisfactory construct validity, indicating the need for further studies with this instrument using another protocol as a reference.

## INTRODUCTION

Hearing allows individuals to connect with the world, as it enables human interaction with the environment, as well as fostering the development of communication and social interaction. However, when an individual is affected by hearing loss, he or she suffers from limitations, even discrimination^(1)^.

The aging process causes several functional and structural changes in the body, which affect the individual's quality of life, and among them, hearing loss stands out^(2)^. This change, inherent to the senescence process, also known as presbycusis, has several implications such as: difficulty to understand speeches, especially in challenging environments (noisy and reverberant), social interaction, depression, and especially social isolation and cognitive decline^(3,4)^. Furthermore, it is also known that the time of hearing deprivation directly affects cognition, causing degradation in the neural system and reducing its functions^(5)^.

There is evidence that the lack of auditory stimulation and the consequent social isolation encourage the loss of cognitive function^(6)^. Several studies indicate that hearing loss may be associated with a higher risk of cognitive decline, Alzheimer's disease and dementia^(7-9)^. Therefore, it is extremely important to assess cognitive processes in the population with hearing loss. However, the instruments available until recently were standardized only for normal-hearing individuals.

The use of formal tests to assess cognition enables an objective analysis of responses, optimizes the performance of correct diagnoses and the definition of more assertive conducts, helping to improve the quality of life of individuals and their families^(10)^. However, it is necessary to use tests that demonstrate the real situation of the subject without interference from other factors, such as low education, depression, hearing loss or delirium^(10)^.

In this sense, the Montreal Cognitive Assessment Hearing Impairment (MoCA-H) cognitive assessment instrument was developed and validated in English through a partnership of researchers from Australia, England, Ireland, Canada, France, Greece and Cyprus, and has proven to be a sensitive and reliable tool for identifying cognitive alterations in elderly individuals with acquired hearing impairment^(11)^. This instrument proposes the presentation of the stimuli/guidelines used in the assessment of cognitive functions, in written format instead of oral presentation^(11)^. In addition to the English version, the MoCA-H is available in Arabic, Chinese, Hungarian, Dutch, German and Italian^(12)^.

In 2023, a study group from southern Brazil carried out the cross-cultural adaptation of the MoCA-H into Brazilian Portuguese (BP), carefully following all the psychometric steps recommended in the literature, namely: translation and back-translation of the MoCA-H, analysis and selection of stimuli, analysis by expert judges, analysis by non-expert judges, and pilot study. It should be noted that the authors of the original instrument followed the development of these steps and agree with the final result of the instrument, which is now available free of charge on the MoCA website for use by duly qualified individuals^(12)^. However, it is extremely important that any instrument subjected to a cross-cultural adaptation be validated in that language, in order to certify that it precisely measures what it is intended to measure^(13)^.

The stages of validity include criterion validity and construct validity. The first measures the degree of effectiveness of a test in predicting a subject's specific performance^(14)^. Obviously, the subject's performance must be measured using techniques other than the test itself that is intended to be validated^(14)^. Construct validity verifies whether the instrument actually measures what it proposes, that is, whether the scores of the measurement are associated with the scores of previously validated constructs^(15)^. In this way, the results of the developed instrument are compared with the results of an already current standard. Validity is achieved by evaluating the scores obtained in the test in question with the scores achieved in the test that will serve as a criterion^(16)^.

Understanding the importance of protocols that assess cognitive aspects of individuals with hearing loss who speak BP, and considering the lack of validation of the recently adapted test, the same researchers who carried out the cross-cultural adaptation process became interested in seeking evidence of the validity of the MoCA-H protocol for this population. It is believed that continuing these steps will contribute significantly to obtaining a reliable instrument that will provide more accurate diagnoses for these individuals, in addition to inspiring new research that can benefit public health and health education.

Therefore, based on what was exposed, the present study aimed to seek evidence of criterion and construct validity for the MoCA-H in BP.

## METHODS

This is an observational, cross-sectional and quantitative study, with a sample selected by convenience from a public Hearing Health Care Service in the interior of Rio Grande do Sul (RS). This study is linked to a project that was approved by the Research Ethics Committee (REC) under No. 5,162,650, and was conducted in accordance with the guidelines and regulatory standards for research involving human beings, as established in Resolution 466/12 of the National Health Council.

### Participants

The following eligibility criteria were established for sample selection: agreement to participate in the study and signing of the Free and Informed Consent Form (FICF); age 60 years or older; Brazilian nationality and fluency in Portuguese; presence of moderate to profound bilateral hearing loss, completion of at least four years of formal education, and preserved or corrected near visual acuity. The exclusion criteria used were the following: presence of self-reported focal neurological injury; previous diagnosis of syndromes; intellectual disability; and non-participation in all procedures proposed in this study.

Thus, 70 elderly individuals participated in the sample and were divided into two groups: G1 - 50 subjects with hearing loss and no cognitive decline; G2 - 20 subjects with hearing loss and cognitive decline. The distribution of subjects between the groups, with regard to the presence or absence of cognitive decline, took into account the performance of the participants in the Mini Mental State Examination (MMSE)^(17)^.

The sample consisted of 43 (61.43%) men and 27 (38.57%) women, aged between 60 and 86 years (M = 70.49 years) and with formal education between 4 and 21 years (M = 6.9 years). Regarding the degree of hearing loss in the right ear, 28.6% of the elderly had moderate hearing loss, 47.1% had moderately severe hearing loss and 24.3% had severe hearing loss. In the left ear, the data showed that 27.1% of the sample had moderate hearing loss, 47.2% had moderately severe hearing loss, 20% had severe hearing loss and 5.7% had profound hearing loss.

The groups were matched by age (p=0.136) and time of formal education (p=0.345) (Table 1).

**Table 1:** Descriptive analysis and comparison of the variables age and time of formal education between the groups

	G1 (N=50)					G2 (N=20)					p-value*
	Min	Max	Median	Mean	SD	Min	Max	Median	Mean	SD	
Age	60	84	68.5	69.7	6.89	61	86	74	72.45	7.24	0.136
Formal study time	4	21	5	7.26	3.65	4	12	5	5.9	1.94	0.345

* Statistical test: Mann-Whitney U test, with a significance level of 5%

Caption: N = sample number; Min = minimum; Max = maximum; SD = standard deviation; G1 = Without cognitive decline; G2 = With cognitive decline

### Procedures

Participants were selected from the aforementioned service and those who were receiving care during the data collection period took part in this study. Initially, the following data were collected: identification data (name, sex and date of birth), education level, nationality and results of the audiological evaluation. All participants signed the informed consent form and underwent anamnesis, pure tone audiometry (PTA) (for those who did not have records of updated evaluations in the period of one year), MMSE and MoCA-H.

The anamnesis consisted of questions aimed at determining whether the participants met the eligibility or exclusion criteria for the research, such as: time of formal study, visual acuity (in the event of a self-reported deficit, questions were asked about correction and frequency of visits to the doctor), presence of neurological alterations, previous diagnosis of syndromes and/or intellectual disability.

Subjects who did not have updated audiological evaluation records underwent an evaluation consisting of inspection of the external auditory canal and PTA. PTA was performed in a soundproof booth, using audiometers of the Resonance R37a Clinical model, from Audiology, and the Ad229e model, from Interacoustics, duly calibrated, coupled to supra-aural TDH-39 headphones.

The MMSE^(17)^ was the procedure used to classify the sample regarding the presence or absence of cognitive decline and thus allocate each participant into one of the groups. The MMSE assesses signs of dementia through tasks of temporal and spatial orientation, immediate memory, attention and calculation, delayed recall, language (naming, repetition, verbal command, reading of written order, written elaboration of sentence) and visual constructive ability (copying of drawing). It has a total of 30 points, with norms based on education, namely: illiterate - 21 points; low education (one to five years) - 22 points; average education (six to 11 years) - 23 points; high education (12 years or more) - 24 points^(18)^.

Subsequently, the MoCA-H(12) was applied, which assesses eight cognitive domains (executive functions, naming, attention, memory, abstract reasoning and orientation, delayed recall, visuospatial skills and language) through the presentation of 77 cards containing instructions and tasks. Participants were required to read each card aloud and follow the instructions contained therein, without any interference from the evaluator. In addition, they received a test sheet and a pen to manually complete the first three tasks. The MoCA-H was applied by duly qualified and certified researchers. Each application lasted, on average, 30 minutes.

It should be noted that when participants did not read the card aloud, the evaluator pointed to the writing until the subject read it orally. Furthermore, when participants asked the evaluator to return to the previous card, she nodded negatively, indicating that this was not allowed.

During the procedures, some adjustments were made in order to avoid the influence of auditory alteration on the cognitive findings of subjects with untreated hearing loss, such as: speech at an adequate intensity, slow and well articulated, in addition to the researchers positioning themselves facing the subject. It is also noteworthy that those who were users of Individual Sound Amplification Devices (ISAD) continued to use the devices throughout the evaluation.

Finally, participants received feedback on their performance in the assessments carried out, and those who obtained altered results in the MMSE were referred to a neurologist, due to the risk of cognitive decline.

### Data analysis

The collected data were tabulated by the researcher in charge in an Excel spreadsheet and subjected to statistical analysis using parametric and non-parametric tests according to the data analyzed. In addition, the data were analyzed descriptively.

Data normality was verified by applying the Shapiro-Wilk test, with a significance level of 5%. The total sample (n=70) was used to analyze criterion validity, which was divided into two groups: G1, composed of subjects with hearing loss and without cognitive decline (n=50); and G2, formed by subjects with hearing loss and cognitive decline (n=20). This analysis aimed to compare the scores obtained in the MoCA-H between the two groups. To this end, the Mann-Whitney U test was used to compare the skill scores between the two groups, and the Student's t-test was used to compare the total MoCA-H scores between the groups. Both tests have a significance level of 5%.

To analyze construct validity, the correlation between total MMSE and MoCA-H scores in subjects with cognitive decline (G2; n=20) was investigated. Given the characteristics of the data analyzed, construct validity was examined using Spearman's correlation test, with a significance level of 5% (p≤0.05). Correlations of up to |0.5| were considered weak; >|0.5| to |0.7|, moderate; and >|0.7|, strong^(19)^.

## RESULTS

When comparing the overall performance and the eight domains assessed in the MoCA-H between subjects with and without cognitive decline, in order to verify the criterion validity of the instrument, it was possible to observe a difference between the groups in the skills of naming, attention, language, abstraction, memory and delayed recall, in addition to the total MoCA-H score, indicating a significantly superior performance for individuals without cognitive decline (Table 2).

**Table 2:** Comparison of overall and skill performance of subjects with and without cognitive decline on the MoCA-H

Description	Group	N	Mean	SD	p-value
MoCA Total	G1	50	19.10	3.759	≥0.000*
	G2	20	14.75	4.411	
Visuospatial/Executive	G1	50	2.32	0.868	0.150
	G2	20	1.85	1.137	
Naming	G1	50	2.58	0.673	0.046**
	G2	20	2.25	0.716	
Attention	G1	50	3.46	1.568	0.004**
	G2	20	2.05	1.877	
Language	G1	50	1.32	0.999	0.015**
	G2	20	0.70	0.801	
Abstraction	G1	50	0.66	0.688	0.033**
	G2	20	0.30	0.571	
Orientation	G1	50	5.18	0.919	0.090
	G2	20	4.75	1.020	
Memory	G1	50	6.98	1.684	0.003**
	G2	20	5.53	1.867	
Late recalling	G1	50	1.78	1.799	0.047**
	G2	20	0.85	1.461	

* Student's t-test (significance level of 5% - p ≤0.05);

** Mann-Whitney U test (significance level of 5% - p ≤0.05)

Caption: SD = standard deviation; MoCA-H = Montreal Cognitive Assessment Hearing Impairment; N = sample number; G1 = subjects with hearing loss and no cognitive decline; G2 = subjects with hearing loss and cognitive decline

When correlating the results of the mean scores of the total MMSE and the total MoCA-H, obtained by the 20 subjects with cognitive decline, with the aim of evaluating the construct validity, a weak and non-significant correlation was found (Rho=0.384; p=0.095) (Table 3).

**Table 3:** Correlation of the total MMSE score with the total MoCA-H score

	N	Mean	SD	Rho*	p-value
MMSE	20	19.85	2.00	0.384	0.095
MoCA-H Total	20	14.60	4.31		

* Statistical test: Spearman correlation, with a significance level of 5% (p≤0.05)

Caption: N = sample number; SD = standard deviation; MoCA-H = Montreal Cognitive Assessment Hearing Impairment; MMSE = Mini Mental State Examination

## DISCUSSION

This study aimed to analyze the psychometric properties of criterion and construct validity of the MoCA-H protocol for elderly Brazilians with moderate to profound hearing loss. For this purpose, the sample consisted of subjects with hearing loss of the mentioned degrees, and all participants with profound hearing loss had a lesser loss in the contralateral ear, which ensured the intelligibility of the procedures. In addition, adjustments were made so that hearing loss did not negatively impact the cognitive assessment, as described in the methodology.

Subjects with unilateral hearing loss or with mild, profound or complete bilateral hearing loss did not participate in the study due to the requirements imposed by the instruments used and the possibility of generating biases. The MoCA-H was developed for elderly individuals with moderate or severe hearing loss, which justified the choice of the initial degree of hearing loss of the participants^(11)^. In turn, individuals with profound and complete bilateral hearing loss were not included due to the application format of the MMSE^(17)^, which is entirely through oral requests and would be directly impacted by hearing loss.

Validation of the protocol studied in each language is extremely important, since variations in the diagnosis of dementia between countries and/or cultural or linguistic differences may be responsible for disparities in protocol performance^(11,20,21)^. Therefore, it is understood that it is not possible to use the same scores from English or German for BP^(11,22)^, as mentioned in validation studies of the standard MoCA.

Since this is the first validation study of the aforementioned instrument in BP, which was conducted in a population diagnosed and rehabilitated in the Brazilian Unified Health System (SUS) and with a relatively short time of formal education (average 6.9 years), comparing the results with the available literature is somewhat challenging, given the scarcity of publications on the subject using the same protocol in a similar population. Furthermore, it is extremely important to highlight that the MoCA-H has been translated and cross-culturally adapted to other languages ​​such as Arabic, Chinese, Hungarian, Dutch, German and Italian^(12)^, however, publications on validation were found in only two languages, English and German.

In this study, the MoCA-H showed satisfactory criterion validity, presenting significant differences between subjects with and without cognitive impairment (Total MoCA-H). In addition, it was possible to observe significant differences between the groups in naming, attention, language, abstraction, memory and delayed recall skills (Table 2). However, no difference was observed in visuospatial-executive and orientation skills. It is assumed that the lack of difference in these skills between subjects with and without cognitive impairment can be attributed to the level of difficulty of the tasks designed to assess them. While the task that assesses visuospatial-executive skills seems very complex to the subjects evaluated, the task that assesses orientation is considered very simple for subjects in both groups. This finding corroborates that found in a study conducted by Tulane University in New Orleans (Louisiana/USA)^(23)^. Therefore, it is suggested that it is relevant to investigate potential changes in the assessment of these skills, aiming to improve their effectiveness and accuracy.

When investigating the criterion validity of the MoCA-H protocol in English, Dawes et al.^(11)^ found differences between the groups for the naming and delayed recall domains, in addition to the mean total score. In the present study, in addition to finding differences in these same skills, differences were found between the groups in four other domains. This suggests that the MoCA-H in BP appears to be more effective in distinguishing between subjects with and without impairment in attention, language, abstraction and memory skills.

Regarding construct validity, a weak and non-significant correlation was obtained between the total scores of the MMSE and MoCA-H protocols (Table 3). It is believed that this finding is justified by the disparity in the levels of demand between the tests, since they have different objectives, the first detecting signs of dementia and the second mild cognitive impairment. This assumption was also mentioned by Nazem et al.^(24)^ in a study carried out with subjects diagnosed with Parkinson's disease. Furthermore, another plausible explanation for the result obtained in this study is the lack of sensitivity and specificity of the MMSE to detect mild cognitive impairment, as previously documented in other studies^(24,25)^.

Furthermore, it is important to highlight that in the MMSE the level of difficulty of the subtests by cognitive domain is different in relation to the MoCA-H, which may have culminated in the occurrence of a ceiling or floor effect in the MMSE in tasks similar to both instruments, contributing to the absence of significant associations. The ceiling effect refers to the achievement of the maximum score in a test by a significant number of participants, while the floor effect occurs when many participants achieve the minimum score. The occurrence of the ceiling effect indicates that the test is not sensitive enough to measure differences between groups with a high level of performance. On the other hand, the occurrence of the floor effect indicates that the test is unable to capture the nuances among those who have very low skills or knowledge.

In contrast, other studies have shown a strong correlation between the MMSE and the MoCA^(26,27)^. However, these studies had samples composed of subjects with a longer period of formal study, which can be considered as a justification for the difference in correlation. However, the MMSE is currently the most viable standardized instrument for application in people with hearing loss. Many studies adopt it as a standard instrument in this population^(25-27)^, due to the scarcity of specific validated protocols on the subject that are quick and easy to apply.

In this study, it was decided not to use the standard MoCA as a validated reference instrument (gold standard), to avoid the effect of learning and facilitation, with improved scores, when applying the MoCA-H, generating bias in the results. According to the literature, studies that do not consider the effect of learning in repeating tests can lead to erroneous conclusions^(28,29)^.

The sample composition by subjects with relatively low formal education time (average 6.9 years) constituted a limitation of the present research, considering the proven impact of education on cognitive performance^(20,30-33)^. Therefore, it is recommended that additional studies be carried out to validate the MoCA-H in BP in samples with longer formal education time.

Furthermore, we highlight the need for further psychometric research seeking evidence of reliability, dependability, sensitivity and specificity of this protocol. Still, we suggest carrying out studies that seek the construct validity of the MoCA-H using another cognitive assessment protocol as a reference, instead of the MMSE.

Finally, it is believed that this instrument will provide more accurate diagnoses in individuals with hearing loss, guiding more assertive behaviors and encouraging the search for treatment in this specific population, contributing to the overall reduction in the incidence of dementia.

## CONCLUSION

The Montreal Cognitive Assessment Hearing Impairment protocol for elderly Brazilians with moderate or greater hearing loss has good criterion validity and is a promising tool for screening for mild cognitive decline. However, further studies are still needed for complete validation, especially with regard to construct validity.
