# Supplementary material for: Montreal Cognitive Assessment Hearing Impairment (MoCA-H) in Brazilian Portuguese: criterion and construct validity
Source: Codas. 2025 Oct 13;37(5):e20240249. doi: 10.1590/2317-1782/e20240249en (PMC12533528; doi:10.1590/2317-1782/e20240249en)
Supplement: Translation

# Translation: Montreal Cognitive Assessment Hearing Impairment (MoCA-H) no Português Brasileiro: validade de critério e de construto

**DOI:** 10.1590/2317-1782/e20240249pt

**Authors:** Gabriela Konrath, Rochele Martins Machado, Karina Carlesso Pagliarin, Fernanda Soares Aurélio Patatt

**Affiliations:** 1 Departamento de Fonoaudiologia, Universidade Federal de Santa Maria – UFSM - Santa Maria (RS), Brasil.

**Keywords:** Audição, Perda Auditiva, Disfunção Cognitiva, Envelhecimento, Presbiacusia, Psicometria, Estudo de Validação, Testes de Estado Mental e Demência

## Abstract

**Objetivo:**

Buscar evidências de validade de critério e de construto para o protocolo Montreal Cognitive Assessment Hearing Impairment (MoCA-H) no Português Brasileiro.

**Método:**

Participaram da amostra 70 idosos distribuídos em dois grupos: Grupo 1-50 sujeitos com perda auditiva e sem declínio cognitivo; Grupo 2-20 sujeitos com perda auditiva e com declínio cognitivo. A validade de critério foi obtida mediante comparação dos Grupos 1 e 2, considerando o escore geral e os oito domínios avaliados no MoCA-H. Os dados foram analisados com o teste U de Mann-Whitney e teste T de Student, respeitando as características dos dados coletados. Para verificação da validade de construto analisou-se a correlação entre os escores totais do Mini Exame do Estado Mental (MEEM) e do MoCA-H obtidos pelo Grupo 2. Para tanto, utilizou-se o Teste de Correlação de Spearman.

**Resultados:**

A análise da validade de critério mostrou diferença entre os grupos com e sem declínio nas habilidades nomeação, atenção, linguagem, abstração, memória e evocação tardia, além do escore total do MoCA-H, indicando desempenho significativamente superior nos sujeitos do Grupo 1. Na análise da validade de construto, foi observado correlação fraca e sem significância (Rho=0,384; p=0,095) entre os escores do MoCA-H e do MEEM.

**Conclusão:**

O protocolo MoCA-H apresentou boa validade de critério para esta população específica, tornando-se uma ferramenta confiável para a triagem de declínio cognitivo leve. No entanto, não apresentou validade de construto satisfatória, indicando a necessidade de mais estudos com o referido instrumento utilizando outro protocolo como referência.

## INTRODUÇÃO

A audição permite conectar os indivíduos ao mundo, visto que ela possibilita a interação do ser humano com o meio ambiente, bem como propicia o desenvolvimento da comunicação e o convívio social. Contudo, quando um indivíduo é acometido pela perda auditiva, ele sofre com limitações, até mesmo discriminações^(1)^.

O processo de envelhecimento ocasiona várias transformações funcionais e estruturais no organismo, que afetam a qualidade de vida do indivíduo e, dentre elas, se destaca a perda auditiva^(2)^. Esta alteração, inerente ao processo de senescência, também conhecida como presbiacusia, acarreta várias implicações como: dificuldade de compreensão de fala, principalmente em ambientes desafiadores (ruidosos e reverberantes), interação social, depressão, e principalmente o isolamento social e o declínio cognitivo^(3,4)^. Ademais, sabe-se ainda, que o tempo de privação auditiva afeta diretamente a cognição, acarretando degradações no sistema neural e reduzindo suas funções^(5)^.

Há evidências que a falta de estimulação auditiva e o consequente isolamento social incitam a perda da função cognitiva^(6)^. Vários estudos apontam que a perda auditiva pode estar associada com um maior risco de declínio cognitivo, doença de Alzheimer e demência^(7-9)^. Sendo assim, é de suma importância a avaliação dos processos cognitivos na população com perda auditiva. No entanto, os instrumentos disponíveis até pouco tempo eram normatizados apenas para indivíduos normo-ouvintes.

O uso de testes formais para avaliar a cognição possibilita uma análise objetiva das respostas, otimiza a realização de diagnósticos corretos e a definição de condutas mais assertivas, auxiliando na qualidade de vida dos indivíduos e seus familiares^(10)^. Entretanto, faz-se necessário utilizar testes que evidenciem a real situação do sujeito sem interferência de outros fatores, como baixa escolaridade, depressão, perda auditiva ou delirium^(10)^.

Neste sentido, foi desenvolvido e validado por meio da parceria de pesquisadores da Austrália, Inglaterra, Irlanda, Canadá, França, Grécia e Chipre, o instrumento de avaliação cognitiva Montreal Cognitive Assessment Hearing Impairment (MoCA-H), na língua inglesa, que se mostrou uma ferramenta sensível e confiável de identificação de alterações cognitivas em idosos com deficiência auditiva adquirida^(11)^. Este instrumento propõe a apresentação dos estímulos/orientações utilizados na avaliação das funções cognitivas, em formato escrito ao invés de apresentação oral^(11)^. Além da versão em inglês, o MoCA-H está disponível em árabe, chinês, húngaro, holandês, alemão e italiano^(12)^.

No ano de 2023 um grupo de estudos do sul do Brasil realizou a adaptação transcultural do MoCA-H para o Português Brasileiro (PB), seguindo criteriosamente todas as etapas psicométricas preconizadas na literatura, a saber: tradução e retrotradução do MoCA-H, análise e seleção dos estímulos, análise de juízes especialistas, análise de juízes não especialistas e estudo piloto. Salienta-se que os autores do instrumento original acompanharam o desenvolvimento das referidas etapas e estão de acordo com o resultado final do instrumento, já disponível gratuitamente, no site do MoCA, para a utilização de pessoas devidamente qualificadas^(12)^. Contudo, é de suma importância que todo instrumento submetido a uma adaptação transcultural seja validado naquela língua, a fim de certificar que ele mensura precisamente o que se propõe a medir^(13)^.

Dentre as etapas de validade encontram-se a validade de critério e a validade de construto. A primeira, mensura o grau de eficácia que um teste tem em predizer o desempenho específico de um sujeito^(14)^. Evidentemente, o desempenho do sujeito deve ser medido por meio de técnicas que não o próprio teste que se quer validar^(14)^. Já a validade de construto, verifica se o instrumento de fato mede o que se propõe, ou seja, se as pontuações da medida se associam com as pontuações de construtos já validados^(15)^. Deste modo, compara-se os resultados do instrumento desenvolvido com os resultados de um padrão já vigente. A validade é atingida pela avaliação dos escores obtidos no teste em questão com os escores alcançados no teste que servirá de critério^(16)^.

Compreendendo a importância da existência de protocolos que avaliem aspectos cognitivos de indivíduos com perda auditiva falantes do PB, e considerando a falta de validação do teste adaptado recentemente, surgiu o interesse, por parte dos mesmos pesquisadores que realizaram o processo de adaptação transcultural, em buscar evidências de validade do protocolo MoCA-H para essa população. Acredita-se que a continuidade dessas etapas contribuirá significativamente para a obtenção de um instrumento confiável que proporcionará diagnósticos mais precisos para esses indivíduos, além de inspirar novas pesquisas que possam beneficiar a saúde pública e a educação em saúde.

Sendo assim, a partir do que foi exposto, o presente estudo teve como objetivo buscar evidências de validade de critério e de construto para o MoCA-H no PB.

## MÉTODO

Trata-se de um estudo de caráter observacional, transversal e quantitativo, com amostra eleita por conveniência em um Serviço de Atenção à Saúde Auditiva público, do interior do Rio Grande do Sul (RS). Este estudo está vinculado a um projeto que foi aprovado pelo Comitê de Ética em Pesquisa (CEP) sob o n° 5.162.650, e foi conduzido de acordo com as diretrizes e normas regulamentadoras para pesquisas envolvendo seres humanos, conforme estabelecido na Resolução 466/12, do Conselho Nacional de Saúde.

### Participantes

Para a seleção da amostra foram estabelecidos os seguintes critérios de elegibilidade: concordância com a participação no estudo e assinatura do Termo de Consentimento Livre e Esclarecido (TCLE); idade igual ou superior a 60 anos; nacionalidade brasileira e fluência em língua portuguesa; presença de perda auditiva bilateral de grau moderado à profundo, conclusão de pelo menos quatro anos de estudo formal e acuidade visual para perto preservada ou corrigida. Quanto aos critérios de exclusão utilizados foram os seguintes: presença de lesão neurológica focal autorrelatada; diagnóstico prévio de síndromes; deficiência intelectual; e não participação em todos os procedimentos propostos neste estudo.

Desta forma, participaram da amostra 70 idosos distribuídos em dois grupos, sendo eles: G1 - 50 sujeitos com perda auditiva e sem declínio cognitivo; G2 - 20 sujeitos com perda auditiva e com declínio cognitivo. Para a distribuição dos sujeitos entre os grupos, no que diz respeito à presença ou não de declínio cognitivo, levou-se em consideração o desempenho dos participantes no Mini Exame de Estado Mental (MEEM)^(17)^.

A amostra foi constituída por 43 (61,43%) homens e 27 (38,57%) mulheres, com idades entre 60 a 86 anos (M=70,49 anos) e com tempo de estudo formal entre 4 e 21 anos (M= 6,9 anos). Em relação ao grau da perda auditiva na orelha direita, 28,6% dos idosos apresentavam perda auditiva moderada, 47,1% apresentavam perda auditiva moderadamente severa e 24,3% tinham perda auditiva severa. Na orelha esquerda, os dados mostraram que 27,1% da amostra tinha perda auditiva moderada, 47,2% apresentava perda moderadamente severa, 20% tinha perda auditiva severa e 5,7% apresentava perda auditiva profunda.

Os grupos foram emparelhados por idade (p=0,136) e tempo de estudo formal (p=0,345) (Tabela 1).

**Tabela 1:** Análise descritiva e comparação das variáveis idade e tempo de estudo formal entre os grupos

	G1 (N=50)					G2 (N=20)					Valor de p*
	Mín	Máx	Mediana	Média	DP	Mín	Máx	Mediana	Média	DP	
Idade	60	84	68,5	69,7	6,89	61	86	74	72,45	7,24	0,136
Tempo de estudo formal	4	21	5	7,26	3,65	4	12	5	5,9	1,94	0,345

* Teste estatístico: teste U de Mann-Whitney, com nível de significância de 5%

Legenda: N = número amostral; Mín = mínimo; Máx = máximo; DP = desvio padrão; G1 = Sem declínio cognitivo; G2 = Com declínio cognitivo

### Procedimentos

Os participantes foram selecionados no serviço supracitado e fizeram parte do presente estudo os que estavam em atendimento no período da coleta dos dados. Inicialmente, foram levantados: dados de identificação (nome, sexo e data de nascimento), escolaridade, nacionalidade e resultados da avaliação audiológica. Todos os participantes assinaram o TCLE e foram submetidos à anamnese, à audiometria tonal liminar (ATL) (para os que não tinham registros de avaliações atualizadas no período de um ano), ao MEEM e ao MoCA-H.

A anamnese foi composta por questões direcionadas para determinar se os participantes atendiam aos critérios de elegibilidade ou exclusão da pesquisa, tais como: tempo de estudo formal, acuidade visual (mediante déficit autorrelatado, questionava-se quanto a correção e a frequência de retorno ao médico), presença de alteração neurológica, diagnóstico prévio de síndromes e/ou deficiência intelectual.

Os sujeitos que não tinham registros atualizados de avaliação audiológica, eram submetidos à avaliação composta por inspeção do meato acústico externo e ATL. A ATL foi realizada em cabina acústica, com uso de audiômetros do modelo Resonance R37a Clinical, da marca Audiology, e do modelo Ad229e, da marca Interacoustics, devidamente calibrados, acoplados a fones supra-aurais TDH-39.

O MEEM^(17)^ foi o procedimento empregado para classificar a amostra com relação à presença ou não de declínio cognitivo e assim alocar cada participante em um dos grupos. O MEEM avalia sinais de demência, por meio de tarefas de orientação temporal e espacial, memória imediata, atenção e cálculo, evocação tardia, linguagem (nomeação, repetição, comando verbal, leitura de ordem escrita, elaboração escrita de frase) e capacidade construtiva visual (cópia do desenho). Dispõe de um total de 30 pontos, com normativas baseadas na escolaridade, a saber: analfabetos - 21 pontos; baixa escolaridade (um a cinco anos) - 22 pontos; média escolaridade (seis a 11 anos) - 23 pontos; alta escolaridade (12 anos ou mais) - 24 pontos^(18)^.

Posteriormente, aplicou-se o MoCA-H^(12)^, que avalia oito domínios cognitivos (funções executivas, nomeação, atenção, memória, raciocínio abstrato e orientação, evocação tardia, habilidades visuo-espaciais e linguagem) por meio da apresentação de 77 cartões contendo as instruções e as tarefas. Os participantes deviam ler em voz alta cada cartão e seguir as orientações contidas neles, sem qualquer interferência da avaliadora. Além disso, recebiam uma folha de teste e uma caneta para resolver manualmente as três primeiras tarefas. O MoCA-H foi aplicado por pesquisadoras devidamente qualificadas e certificadas. Cada aplicação durou, em média, 30 minutos.

Salienta-se que quando os participantes não realizavam a leitura do cartão em voz alta, a avaliadora apontava para a escrita até que o sujeito realizasse a leitura oral. Ainda, quando os participantes solicitavam à avaliadora que retornasse ao cartão anterior, a mesma sinalizava negativamente com a cabeça, indicando que isso não era permitido.

Durante os procedimentos foram realizados alguns ajustes com o intuito de não haver influência da alteração auditiva nos achados cognitivos dos sujeitos com perda auditiva não tratada, sendo estes: fala em intensidade adequada, pausada e bem articulada, além das pesquisadoras posicionarem-se de frente para o sujeito. Destaca-se também que aqueles que eram usuários de Aparelhos de Amplificação Sonora Individual (AASI) mantinham o uso dos dispositivos durante toda a avaliação.

Por fim, os participantes receberam a devolutiva do seu desempenho nas avaliações realizadas, e aqueles que obtiveram resultado alterado no MEEM receberam encaminhamento para o médico neurologista, devido ao risco de declínio cognitivo.

### Análise dos dados

Os dados coletados foram tabulados pela pesquisadora responsável, em planilha do Excel, e submetidos a análise estatística com utilização de testes paramétricos e não paramétricos de acordo com os dados analisados. Além disso, os dados foram analisados descritivamente.

A normalidade dos dados foi verificada por meio da aplicação do teste de Shapiro-Wilk, com nível de significância de 5%. Para a análise da validade de critério utilizou-se a amostra total (n=70), que foi dividida em dois grupos: G1, composto por sujeitos com perda auditiva e sem declínio cognitivo (n=50); e G2, formado por sujeitos com perda auditiva e com declínio cognitivo (n=20). Esta análise teve como intuito comparar os escores obtidos no MoCA-H entre os dois grupos. Para tanto, empregou-se o teste U de Mann-Whitney para comparar os escores das habilidades entre os dois grupos, e o teste T de Student para comparar os escores do MoCA-H total entre os grupos. Ambos os testes tem um nível de significância de 5%.

Para a análise da validade de construto, investigou-se a correlação entre os escores totais do MEEM e do MoCA-H nos sujeitos com declínio cognitivo (G2; n=20). Dadas as características dos dados analisados, a validade de construto foi examinada utilizando o Teste de Correlação de Spearman, com um nível de significância de 5% (p≤0,05). Correlações de até |0,5| foram consideradas fracas; >|0,5| a |0,7|, moderadas; e >|0,7|, fortes^(19)^.

## RESULTADOS

Ao comparar o desempenho geral e os oito domínios avaliados no MoCA-H entre os sujeitos com e sem declínio cognitivo, a fim de verificar a validade de critério do instrumento, pode-se constatar diferença entre os grupos nas habilidades de nomeação, atenção, linguagem, abstração, memória e evocação tardia, além do escore total do MoCA-H, indicando um desempenho significativamente superior para os indivíduos sem declínio cognitivo (Tabela 2).

**Tabela 2:** Comparação do desempenho geral e por habilidades dos sujeitos com e sem declínio cognitivo no MoCA-H

Descrição	Grupo	N	Média	DP	Valor de p
MoCA Total	G1	50	19,10	3,759	≥0,000*
	G2	20	14,75	4,411	
Visuoespacial/Executiva	G1	50	2,32	0,868	0,150
	G2	20	1,85	1,137	
Nomeação	G1	50	2,58	0,673	0,046**
	G2	20	2,25	0,716	
Atenção	G1	50	3,46	1,568	0,004**
	G2	20	2,05	1,877	
Linguagem	G1	50	1,32	0,999	0,015**
	G2	20	0,70	0,801	
Abstração	G1	50	0,66	0,688	0,033**
	G2	20	0,30	0,571	
Orientação	G1	50	5,18	0,919	0,090
	G2	20	4,75	1,020	
Memória	G1	50	6,98	1,684	0.003**
	G2	20	5,53	1.867	
Evocação Tardia	G1	50	1,78	1,799	0,047**
	G2	20	0,85	1,461	

* Teste estatístico T de Student (nível de significância de 5% - p ≤0,05);

** Teste U de Mann-Whitney (nível de significância de 5% - p ≤0,05)

Legenda: DP = desvio padrão; MoCA-H = Montreal Cognitive Assessment Hearing Impairment; N = número amostral; G1 = sujeitos com perda auditiva e sem declínio cognitivo; G2 = sujeitos com perda auditiva e com declínio cognitivo

Ao correlacionar os resultados dos escores médios do MEEM total e do MoCA-H total, obtidos pelos 20 sujeitos com declínio cognitivo, com intuito de avaliar a validade de construto, constatou-se correlação fraca e não significativa (Rho=0,384; p=0,095) (Tabela 3).

**Tabela 3:** Correlação do escore total do MEEM com escore total do MoCA-H

	N	Média	DP	Rho*	Valor de p
MEEM	20	19,85	2,00	0,384	0,095
MoCA-H Total	20	14,60	4,31		

* Teste estatístico: Correlação de Spearman, com nível de significância de 5% (p≤0,05)

Legenda: N = número amostral; DP = desvio padrão; MoCA-H = Montreal Cognitive Assessment Hearing Impairment; MEEM = Mini

Exame do Estado Mental

## DISCUSSÃO

Neste estudo, buscou-se analisar as propriedades psicométricas de validade de critério e construto do protocolo MoCA-H para idosos brasileiros com perda auditiva de grau moderado a profundo. Para isso, a amostra foi composta por sujeitos com perda auditiva desses graus, sendo que todos os participantes com perda auditiva profunda apresentavam uma perda menor na orelha contralateral, o que garantiu a inteligibilidade dos procedimentos. Além disso, foram realizados ajustes para que a perda auditiva não impactasse negativamente na avaliação cognitiva, conforme descrito nos métodos.

Sujeitos com perda auditiva unilateral ou com perda auditiva bilateral leve, profunda ou completa não participaram da pesquisa devido às exigências impostas pelos instrumentos utilizados e possibilidades de gerar vieses. O MoCA-H foi desenvolvido para idosos com perda auditiva de grau moderado ou superior, o que justificou a escolha do grau inicial da perda auditiva dos participantes^(11)^. Por sua vez, indivíduos com perdas auditivas profunda e completa bilateral não foram incluídos em razão do formato de aplicação do MEEM^(17)^, que é totalmente por meio de solicitações orais e seria diretamente impactado pela perda auditiva.

A validação do protocolo estudado em cada língua é de extrema importância, visto que, variações no diagnóstico de demência entre países e/ou diferenças culturais ou linguísticas podem ser responsáveis ​​por disparidades no desempenho do protocolo^(11,20,21)^. Desse modo, entende-se não ser possível utilizar os mesmos escores do inglês ou do alemão para o PB^(11,22)^, bem como mencionado em estudos de validação do MoCA padrão.

Em virtude deste ser o primeiro estudo de validação do referido instrumento no PB, que foi conduzido em uma população diagnosticada e reabilitada no Sistema Único de Saúde (SUS) e com tempo de estudo formal relativamente baixo (média 6,9 anos), comparar os resultados com a literatura disponível é um tanto desafiador, visto a escassez de publicações sobre a temática utilizando o mesmo protocolo em população semelhante. Além do mais, é de suma importância destacar que o MoCA-H foi traduzido e adaptado transculturalmente para outras línguas como árabe, chinês, húngaro, holandês, alemão e italiano^(12)^, porém foram encontradas publicações sobre validação em apenas duas línguas, inglês e alemão.

Na presente pesquisa o MoCA-H mostrou validade de critério satisfatória, apresentando diferenças significativas entre sujeitos com e sem alteração cognitiva (MoCA-H Total). Além disso, foi possível observar diferença significativa entre os grupos nas habilidades de nomeação, atenção, linguagem, abstração, memória e evocação tardia (Tabela 2). No entanto, não foi constatada diferença nas habilidades visuoespacial-executiva e orientação. Pressupõe-se que a ausência de diferença nas referidas habilidades entre sujeitos com e sem alteração cognitiva, possa ser atribuída ao nível de dificuldade das tarefas destinadas a avaliá-las. Enquanto a tarefa que avalia a habilidade visuoespacial-executiva parece muito complexa aos sujeitos avaliados, a tarefa que avalia orientação é considerada muito simples, para os sujeitos de ambos os grupos. Este achado corrobora o encontrado em estudo realizado pela Universidade de Tulane em Nova Orleans (Luisiana/EUA)^(23)^. Assim, sugere-se que seja relevante investigar potenciais alterações na avaliação dessas habilidades, visando aprimorar sua eficácia e precisão.

Ao investigar a validade de critério no protocolo MoCA-H em inglês, Dawes et al.^(11)^, constataram diferença entre os grupos para os domínios nomeação e evocação tardia, além da pontuação total média. Já no presente estudo, além de ser constatada diferença nessas mesmas habilidades, evidenciou-se diferença entre os grupos em outros quatro domínios. Isso sugere que o MoCA-H no PB parece ser mais eficaz na distinção entre sujeitos com e sem comprometimento nas habilidades de atenção, linguagem, abstração e memória.

Em relação a validade de construto, obteve-se uma correlação fraca e sem significância entre os escores totais dos protocolos MEEM e MoCA-H (Tabela 3). Acredita-se que este achado se justifica pela disparidade nos níveis de exigência entre os testes, visto que eles têm objetivos distintos, o primeiro detecta sinais de demência e o segundo comprometimento cognitivo leve. Esse pressuposto também foi referido por Nazem et al.^(24)^ em estudo realizado com sujeitos diagnosticados com doença de Parkinson. Ainda, outra explicação plausível para o resultado obtido neste estudo é a carência de sensibilidade e especificidade do MEEM para detectar comprometimento cognitivo leve, como previamente documentado em outras pesquisas^(24,25)^. Ademais, é importante salientar que no MEEM o nível de dificuldade dos subtestes por domínio cognitivo é diferente em relação ao MoCA-H o que pode ter culminado na ocorrência de efeito teto ou chão no MEEM em tarefas semelhantes a ambos os instrumentos, contribuindo para a ausência de associações significativas. O efeito teto refere-se à obtenção da pontuação máxima em um teste por um número significativo de participantes, enquanto o efeito chão ocorre quando muitos participantes atingem a pontuação mínima. A ocorrência do efeito teto indica que o teste não é sensível o suficiente para medir diferenças entre grupos com alto nível de desempenho. Por outro lado, a ocorrência do efeito chão indica que o teste não consegue capturar as nuances entre aqueles que possuem habilidades ou conhecimentos muito baixos.

Em contrapartida, outros estudos evidenciaram uma forte correlação entre o MEEM e o MoCA^(26,27)^. No entanto, essas pesquisas tinham amostras compostas por sujeitos com tempo de estudo formal maior, o que pode ser considerado como uma justificativa para a diferença na correlação. Entretanto, o MEEM é atualmente o instrumento normatizado mais viável para aplicação em pessoas com perda auditiva. Muitos estudos o adotam como instrumento padrão nessa população^(25-27)^, devido à escassez de protocolos validados específicos sobre o tema, de rápida e fácil aplicação.

Nessa pesquisa optou-se por não utilizar o MoCA padrão como instrumento validado de referência (padrão-ouro), para evitar o efeito da aprendizagem e facilitação, com melhora dos escores, ao aplicar o MoCA-H, gerando viés nos resultados. Segundo a literatura, os estudos que não consideram o efeito da aprendizagem na repetição dos testes podem levar a conclusões equivocadas^(28,29)^.

A composição da amostra por sujeitos com tempo de estudo formal relativamente baixo (média 6,9 anos) configurou uma limitação da presente pesquisa, considerando o impacto comprovado da escolaridade no desempenho cognitivo^(20,30-33)^. Assim, recomenda-se a realização de estudos adicionais para validar o MoCA-H no PB em amostras com tempo de estudo formal maior.

Ainda, destaca-se a necessidade de mais pesquisas psicométricas buscando evidências de confiabilidade, fidedignidade, sensibilidade e especificidade do referido protocolo. Além disso, sugere-se a realização de estudos que busquem a validade de construto do MoCA-H utilizando outro protocolo de avaliação cognitiva como referência, ao invés do MEEM.

Por fim, acredita-se que este instrumento irá proporcionar diagnósticos mais precisos em indivíduos com perda auditiva, norteando condutas mais assertivas e incentivando a busca por tratamento nessa população específica, contribuindo para a redução global da incidência de demência.

## CONCLUSÃO

O protocolo Montreal Cognitive Assessment Hearing Impairment para idosos brasileiros com perda auditiva moderada ou superior, possui boa validade de critério, apresentando-se como uma ferramenta promissora para triagem de declínio cognitivo leve. Entretanto, ainda necessita de mais estudos para uma validação completa, especialmente no que se refere à validade de construto.
